# Sox-2 Positive Neural Progenitors in the Primate Striatum Undergo Dynamic Changes after Dopamine Denervation

**DOI:** 10.1371/journal.pone.0066377

**Published:** 2013-06-18

**Authors:** Cristina Ordoñez, Paz Moreno-Murciano, Maria Hernandez, Carla Di Caudo, Iñaki Carril-Mundiñano, Nerea Vazquez, Jose Manuel Garcia-Verdugo, Rosario Sanchez-Pernaute, Maria-Rosario Luquin

**Affiliations:** 1 Laboratory of Regenerative Therapy, Neuroscience Division, Centro de Investigación Médica Aplicada and Department of Neurology, Clínica Universidad de Navarra, Pamplona, Spain; 2 Laboratory of Cellular Morphology, Centro de Investigación Principe Felipe, CIBERNED, Valencia, Spain; 3 Laboratory of Stem Cells and Neural Repair, Fundacion Inbiomed, San Sebastian, Spain; 4 Laboratory of Comparative Neurobiology, Instituto Cavanilles, Universidad de Valencia, CIBERNED, Paterna, Spain; Florey Institute of Neuroscience & Mental Health, Australia

## Abstract

The existence of endogenous neural progenitors in the nigrostriatal system could represent a powerful tool for restorative therapies in Parkinson's disease. Sox-2 is a transcription factor expressed in pluripotent and adult stem cells, including neural progenitors. In the adult brain Sox-2 is expressed in the neurogenic niches. There is also widespread expression of Sox-2 in other brain regions, although the neurogenic potential outside the niches is uncertain. Here, we analyzed the presence of Sox-2^+^ cells in the adult primate (*Macaca fascicularis)* brain in naïve animals (N = 3) and in animals exposed to systemic administration of 1-methyl-4-phenyl-1,2,3,6 tetrahydropyridine to render them parkinsonian (N = 8). Animals received bromodeoxyuridine (100 mg/kg once a day during five consecutive days) to label proliferating cells and their progeny. Using confocal and electron microscopy we analyzed the Sox-2^+^ cell population in the nigrostriatal system and investigated changes in the number, proliferation and neurogenic potential of Sox-2^+^ cells, in control conditions and at two time points after MPTP administration. We found Sox-2^+^ cells with self-renewal capacity in both the striatum and the substantia nigra. Importantly, only in the striatum Sox-2^+^ was expressed in some calretinin^+^ neurons. MPTP administration led to an increase in the proliferation of striatal Sox-2^+^ cells and to an acute, concomitant decrease in the percentage of Sox-2^+^/calretinin^+^ neurons, which recovered by 18 months. Given their potential capacity to differentiate into neurons and their responsiveness to dopamine neurotoxic insults, striatal Sox-2^+^ cells represent good candidates to harness endogenous repair mechanisms for regenerative approaches in Parkinson's disease.

## Introduction

Parkinson's disease is a neurodegenerative disorder characterized by a progressive degeneration of the nigral dopamine neurons. The neuronal loss produces a reduction of striatal levels of dopamine resulting in motor dysfunction. Pharmacological restoration of dopamine levels alleviates the cardinal symptoms of the disorder but several motor complications appear with chronic replacement treatment. This fact has led to a search for alternative treatments, including cellular therapy. In addition to ethical and logistic problems, fetal cell transplantation has been only moderately successful and, in some instances, complicated by adverse effects [1], [2], [3]. An attractive alternative would be to direct the neurogenic potential of the adult human brain to restore the nigrostriatal function. In this regard, it is well established that neurogenesis persists in the adult mammalian brain in the subventricular zone (SVZ) of the lateral ventricles and the subgranular zone (SGZ) of the dentate gyrus (for review see [4]). Whether adult neurogenesis takes place in other regions [5], [6], [7] is less clear. In rodents, cells with neurogenic potential have been isolated from the cortex, striatum, spinal cord and substantia nigra (SN) [8], [9], [10], [11], [12], [13]. These cells have self-renewal capacity *in vitro* and are able to give rise to both neuronal and glial lineages [8], [9], [10], [11], [12], [13]. Interestingly, a population of actively dividing progenitor cells has been described in the SN of the adult rat brain [8]. These cells did not give rise to neurons *in vivo* but acquired the capacity to generate new neurons after being transplanted into the hippocampus, a neurogenic area [8]. Moreover, it has been reported that local progenitors generate new neurons in the striatum of adult rat and rabbit [14], [15]. Finally, an increase in neurons has been found to take place in the striatum and cortex in response to certain insults in rodents and primates [16], [17], [18], [19], [20], [21]. Together, these studies suggest that, in these regions, there are quiescent neural precursors capable of generating new neurons when activated by specific factors.

Sox-2 is a sex-determining region Y (SRY) - box2 - related transcription factor expressed during development in self-renewing and multipotent neuroepithelial stem cells [22], [23]. Sox-2 maintains neural progenitor identity and inhibits neuronal differentiation by repressing basic helix-loop-helix (bHLH) proneurogenic factors [24], [25], [26]. Undifferentiated and multipotent cells express Sox-2 and its expression needs to be down-regulated for the cells to acquire mature phenotypes [24], [25]. Sox-2^+^ cells isolated from the adult mouse brain form spheres *in vitro* and can differentiate into neurons, astrocytes and oligodendrocytes by changing culture conditions [27]. Genetic lineage tracing and transplantation experiments in mice have shown that Sox-2-expressing cells continuously give rise to mature cells in the dentate gyrus [28]. In other organs, Sox-2^+^ cells display self-renewal and differentiation potential to maintain tissue homeostasis [29]. Sox-2 is considered a marker of quiescent and amplifying progenitors in the neurogenic niches [30], [31], [32], but it is also expressed in non-neurogenic areas of the rodent brain such as the cortex and the striatum [31]. However, according to that study [31] Sox-2^+^ cells in non-neurogenic areas of the rodent brain do not divide and they all express glial fibrillary acidic protein (GFAP). Nevertheless, in another recent study, new striatal neurons could be generated from Sox-2^+^ precursors in a lesion model of striatal degeneration [33].

Expression of Sox-2 in cells of the SVZ and the SGZ of non-human primates has been previously reported [34], [35] but there are no available data concerning its expression in non-neurogenic regions of the adult brain. In this study, we have analyzed the expression of Sox-2 in the nigrostriatal system in macaques *(Macaca fascicularis)*, to examine their presence in these structures, their capability to generate new neurons and their response to neurotoxicity and dopamine loss in a model of Parkinson's disease.

## Materials and Methods

### Animals

Eleven adult (4–5 years), male monkeys (*Macaca fascicularis*) weighing 3–5 kg were included in the study. They were housed under standard conditions of air exchange (16 l/min), humidity (50%), and light/night cycles (8 a.m. to 8 p.m.), and were fed fresh fruit and commercial pellets, with free access to water. Their health was monitored by the attending veterinarian, in consistency with the recommendations of the Weatherall Report. The animals were euthanized following deep anaesthesia, and all efforts were made to minimize suffering. Experimental protocol was in accordance with the European Communities Council Directive of 24 November 1986 (86/609/EEC) and was approved by the Ethics Committee for Animal Experimentation of the University of Navarra.

Eight monkeys received a weekly intravenous injection of 1-methyl-4-phenyl-1,2,3,6 tetrahydropyridine (MPTP) (Sigma) (0. 25 mg/Kg) during eight consecutive weeks. The cumulative MPTP dose was 2 mg/Kg. Three animals were not treated with MPTP and served as controls. The 3 control animals and 4 MPTP-treated animals were given an intraperitoneal injection of bromodeoxyuridine (BrdU) (Sigma), 100 mg/Kg/day for 5 consecutive days starting one week after the last MPTP injection, and were sacrificed 3 months later (short-term MPTP group n = 4, and control group n = 3). The 3 months interval after BrdU was chosen based on previous works [36], [37] that have established that this period is necessary for detecting newborn and mature neurons in the monkey brain. The other 4 MPTP treated animals were sacrificed 18 months after the last MPTP injection (long-term MPTP group n = 4). The 18 months interval was chosen to assess the specific effect of the reduction in dopamine levels at a chronic stage once the reaction to the toxic insult is over.

### Immunohistochemistry

Animals were deeply anesthetized and transcardially perfused with saline followed by 4% paraformaldehyde in phosphate-buffered saline (PBS). Brains were post-fixed overnight in the same fixative at 4°C and cryoprotected in a 30% sucrose solution in PBS. Coronal sections (40 µm thick) were cut on a freezing microtome, collected in anti-freezing solution (0.125 M PBS, 15% dimethylsulfoxide, and 15% glycerin) and stored at -20°C for later analysis. All analyses were carried out on the right hemisphere. All immunohistochemical assays were performed in free-floating sections. Rat embryonic tissue was used as a positive control for Sox-2. Omission of the primary antibody served as a negative control.

Coronal sections containing striatum, SVZ, SN *pars compacta* and dentate gyrus, were processed for immunohistochemical studies. Sections were washed with PBS, treated with 3% H_2_O_2_ with 10% methanol for 15 minutes, blocked for nonspecific binding with donkey serum (1/20) and incubated with an antibody raised against Sox-2 (goat IgG; 1/200; Neuromics) or tyrosine hydroxylase (TH) (1/1000; Millipore) for 24 hours at 4°C in PBS containing 0.1% Triton X-100 (Sigma Chemical Co). Subsequently, they were incubated with a biotinylated anti-goat secondary antibody (1/500, Jackson Immunoresearch) for 30 minutes and processed with an avidin-biotin complex kit (Vectastain ABC kit; Vector Laboratories) for 30 minutes at room temperature. The chromogen solution was 3′, 3′-diaminobenzidine (DAB) with H_2_O_2_ (DAB kit, Vector Laboratories). Sections were rinsed in distilled water and 0.01 M PBS, mounted on gelatin-coated slides and air-dried, counterstained with Nissl, coverslipped using DPX (BDH) and examined by light microscopy.

### Immunofluorescence Techniques

Double and triple-immunofluorescence techniques were performed in the SVZ, anterior striatum, dentate gyrus and SN sections to detect cells double-labeled with Sox-2 and markers of cell proliferation and differentiation. The primary antibodies used were goat anti-Sox-2 (1/200; Neuromics and R&D Systems); rat anti-BrdU (1/100; Abcam); rabbit anti-Ki67 (prediluted; Master Diagnostica); rabbit anti-GFAP (1/500; Dako); guinea pig anti-doublecortin (DCX) (1/100; Millipore); rabbit anti-calretinin (CR) (1/200; Millipore); mouse anti-NeuN (1/500; Millipore); mouse anti-TH (1/1000; Millipore); rabbit anti-TH (1/1000; Millipore) and mouse anti-glutamic acid decarboxylase 67 (GAD-67) (1/500; Millipore). For BrdU^+^ cell detection, sections were pretreated with 2 N hydrochloric acid at 37°C for 30 minutes and 0.1 M boric acid at room temperature for 15 minutes. For Ki-67^+^ cell detection, sections were pretreated with 10 mM citrate buffer pH 6 at 80°C for 30 min. Secondary antibodies are listed in Table 1.

**Table 1:** List of the secondary antibodies used in the present study.

Antibody	Host	Source, catalog #	Working concentration
-Biotin SP conjugatedDonkey anti-rat	Donkey	JacksonInmmunoresearch 712065153	1∶500
-Donkey anti-goat 488	Donkey	Molecular probes A11055	1∶500
-Donkey anti-goat 568	Donkey	Molecular probes A11057	1∶500
-Donkey anti-goat 633	Donkey	Molecular probes A21082	1∶500
-Donkey anti-mouse 488	Donkey	Molecular probes A21209	1∶500
-Donkey anti-rabbit 555	Donkey	Molecular probes A31572	1∶500
-Donkey anti-rabbit 647	Donkey	Molecular probes A31573	1∶500

A standard immunohistochemical procedure was followed. After incubation overnight at 4°C with a mixture of primary antibodies in PBS containing 0.1% Triton X-100, sections were washed 3 times in PBS for 5 minutes and incubated with the fluorophore-conjugated specific secondary antibodies for 2 hours at room temperature. Some sections were incubated with To-pro3 for 10 minutes. All sections were mounted with 1% PBS-Glycerol and examined under a confocal microscope (LSM 510 Meta, Zeiss).

### Electron Microscopy

To analyze the ultrastructural features of Sox-2^+^ cells we used a pre-embedding DAB immuno-staining (as above) and a pre-embedding immuno-gold technique. For immuno-gold technique, coronal sections (40 µm) were washed in 0.1 M PBS and freeze-thawed (3x) in methyl-butane. Sections were washed in PBS, blocked in 0.3% bovine serum albumin-C (BSA) and incubated in primary goat anti-Sox-2 antibody (AF2018, R&D System) 1/50 in blocked buffer for 3 days at 4°C. Sections were washed in PBS, blocked in 0.5% BSA and 0.1% fish gelatin during 1 hour, incubated in colloidal gold conjugated rabbit anti-goat secondary antibody (1/50) for 24 hours. Silver enhancement was performed and washed again in 2% sodium acetate, washed with gold chloride 0.05%, washed in sodium thiosulfate 0.3% then washed in PBS and post-fixed in 2% glutaraldehyde (30 min) [38]. Sections were contrasted with 1% osmium and 7% glucose and embedded in araldite. Semi-thin 1.5 µm sections were prepared and the section(s) of interest were selected under light microscopy and re-embedded for ultra-thin sectioning at 70 nm. Photomicrographs were obtained under a transmission electron microscope (FEI Tecnai G2 Spirit BioTwin) using a digital camera (Morada, Soft Imaging System, Olympus).

### Quantifications

The estimation of the total number of TH^+^ neurons in the SN was done by stereology using an Olympus BX-51 microscope hard-coupled to a Prior H128 computer-controlled x-y-z motorized stage, a DP70 camera and a computer equipped with Olympus CAST system version 2.0 (Olympus, Albertslund). The analyzed regions were outlined under a low magnification (UPlanApo 4x/0.16, Olympus) objective at all levels in the rostrocaudal axis (6–7 sections per animal). A counting frame was randomly superimposed on the image and neurons were systematically sampled using a 60x lens (Plan Apo N 60x/1.42 oil, Olympus) with the nucleolus used as the sampling unit. The sampling frequency was chosen by adjusting the xy-axis step length so that up to 200 cells were counted in each specimen.

In the striatum, only TH^+^ structures with a visible Nissl-labeled nucleus were counted, to ensure that they were indeed cells. Cell counting was undertaken using bright-field microscopy under a 20× objective lens (Olympus BX51) in four brain sections, taken at similar anatomical levels among animals of the anterior striatum (AP: from +1 to +5 mm according to the atlas of Martin-Bowden [39]. In each animal, cell density (number of TH^+^ neurons per cm^2^) was calculated by dividing the total number of TH^+^ neurons by the area.

Estimation of Sox-2^+^ cellular density was done by stereology in the striatum, SN and SGZ of the dentate gyrus as described above for TH^+^ neurons but cells were sampled using a 20x lens (PlanApo 20x/0.70, Olympus). In each animal, the Sox-2 cell density was obtained by dividing the total number of Sox-2^+^ cells by the total analyzed area (mm^2^).

Sox-2^+^/BrdU^+^ cells in the striatum were counted using a laser scanning confocal microscope (LSM 510/Meta, Zeiss, Germany) under a 20x objective lens in three brain sections, taken at equivalent anatomical levels of the anterior striatum (AP: from +1 to +5 mm according to the atlas of Martin-Bowden, (1996) [39]. In each animal, cell density (number of Sox^+^/BrdU^+^ cells per mm^2^) was calculated by dividing the total number of Sox^+^/BrdU^+^ cells by the surveyed area.

To calculate the percentage of CR**^+^** cells that expressed Sox-2 we used three matched sections of the pre-commisural striatum of each animal (AP: from +1 to +5 mm according to the atlas of Martin-Bowden, (1996) [39]). At least 500 CR**^+^** cells distributed within the entire section were analyzed for each animal, and double-labeled cells were identified using a confocal microscope. For statistical comparisons between groups we used the number of CR**^+^** cells that expressed Sox-2 over the total number of CR**^+^** cells (%).

### Western Blot Analysis

Dissection of the anterior striatum was carried out in ten 40 µm sections for each animal. For protein extraction from formalin fixed tissues,100 µL of 20 mM Tris-HCl buffer pH 9 containing 2% SDS was added and then they were heated at 100°C on a heat block (VWR Scientific Products) for 20 min, and at 60°C in a heater (Indelab) for 2 hours [40]. Samples (10 µg) were separated by electrophoresis SDS-PAGE in a Bio-Rad Miniprotean III electrophoresis unit. Subsequently, proteins were electrotransferred onto a polyvinylide fluoride sheets (PVDF) membrane (Bio-Rad). Membranes were blocked with Tris-NaCl-Tween 20 (TNT) with 5% non-fat milk for 1 hour at room temperature and incubated with primary antibodies against TH (1/2000) (Millipore) overnight at 4°C. After three washes of 10 min each in TNT, membranes were incubated for 1 hour at room temperature with a horseradish-peroxidase conjugated secondary antibody (GE healthcare). Actin was employed as a control. Proteins were detected by chemiluminescence using ECL reagent (Thermo scientific) according to the manufacturer’s instructions on a Kodak X-Omat film.

### Schematic Drawings of Cell Distribution for Calretinin^+^/Sox-2^+^ Double-labeled Cells and TH^+^ Cells

Tissue sections were examined under an Olympus BX-51 microscope hard-coupled to a DP70 camera and a computer equipped with Olympus CAST system version 2.0 (Olympus, Albertslund).

TH^+^ neurons in DAB stained preparations and double labeled Calretinin^+^/Sox-2^+^ in double-immunofluroscence stained sections were systematically sampled using a 60× lens (Plan Apo N 60×/1.42 oil, Olympus) and marked on the screen using the software Olympus CAST system version 2.0 (Olympus, Albertslund). The same sections were photomicrographed using a 4× lens (UPlanApo 4×/0.16, Olympus) objective. The acquired images were assembled in Adobe Photoshop CS to generate pictures of the whole striatum. The contours and marks on all figures were drawn using Macromedia FreeHand MXa.

### Statistical Analysis

Given the small sample size, non-parametric tests were applied and data are given as median and quartiles. For multiple comparisons, a non-parametric Kruskal-Wallis test was used to estimate the overall significance followed by a non-parametric Mann-Whitney *U* test for independent groups with significance defined as p≤0.05.

## Results

### Localization and Characterization of Sox-2^+^ Cells in the Intact Nigrostriatal System of Adult Primates

**Figure 1:**
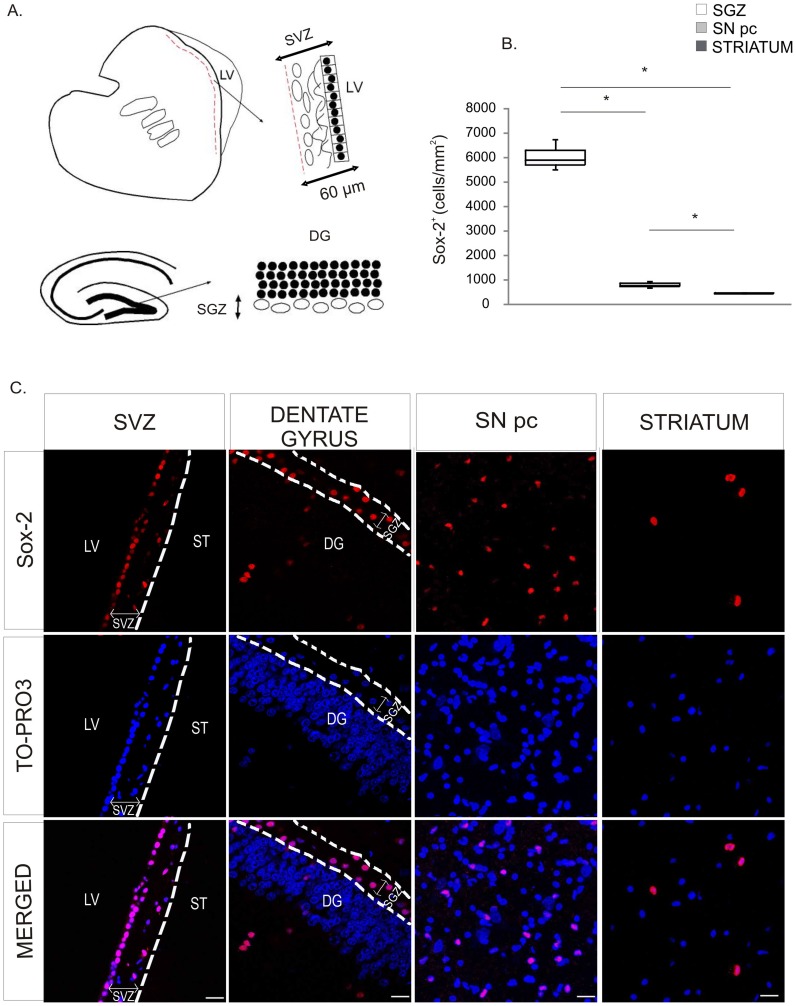
Expression of Sox-2 in neurogenic niches and the nigrostriatal system in the adult primate brain. **A.** Schematic outline of the anatomical borders for the neurogenic niches as defined for the study. **B.** Confocal images of Sox-2^+^ cells in the neurogenic niches and nigrostriatal regions. Sox-2^+^ cells were evenly distributed throughout the brain parenchyma including the striatum and the SNpc. **C.** Stereological estimation of Sox-2^+^ cells revealed that the density of Sox-2^+^ cells was higher in the SNpc than in the striatum and lower in both regions than in the neurogenic niches (data shown for SGZ). Data represent median and quartiles, N = 3, *p≤0.05. Scale bar = 20 µm. Abbreviations: Substantia nigra pars compacta: SNpc; subgranular zone: SGZ.

We first examined the presence of Sox-2^+^ cells in neurogenic niches and in the nigrostriatal system (Fig. 1). Immunohistochemical analysis revealed that, as previously described [34], [35] Sox-2 nuclear immuno-reactivity was present in the SVZ and in the SGZ of the dentate gyrus (Fig. 1A,C). In the SVZ, Sox-2^+^ cells were mainly located in the ependymal layer and in the astrocytic ribbon layer (cellular layer). In the dentate gyrus, Sox-2^+^ cells were predominantly situated within the SGZ and there were some Sox-2^+^ cells in the granular and molecular layers. Interestingly, we also found that Sox-2^+^ cells were broadly distributed within the brain parenchyma in non-neurogenic areas, including the striatum and the SN (Fig. 1C). The specificity of the staining in these regions was confirmed for both secondary antibodies (Fig. S1A). Like in the neurogenic regions the Sox-2 staining was nuclear (Fig. S1B). Outside the neurogenic niches, Sox-2^+^ cells were spread throughout the parenchyma and therefore Sox-2^+^ cell density was considerably lower in the striatum and the SN than in the SGZ of the dentate gyrus (SGZ of the dentate gyrus: Q1∶5715; median: 5898; Q3∶6320 cells/mm^2^, *vs* SN: Q1∶740; median: 783; Q3∶862 cells/mm^2^, p = 0.05; striatum: Q1∶424; median: 436; Q3∶458 cells/mm^2^, p = 0.05). The density of Sox-2^+^ cells was significantly higher in the SN than in the striatum (p = 0.05) (Fig. 1B and Fig. S1C).

**Figure 2:**
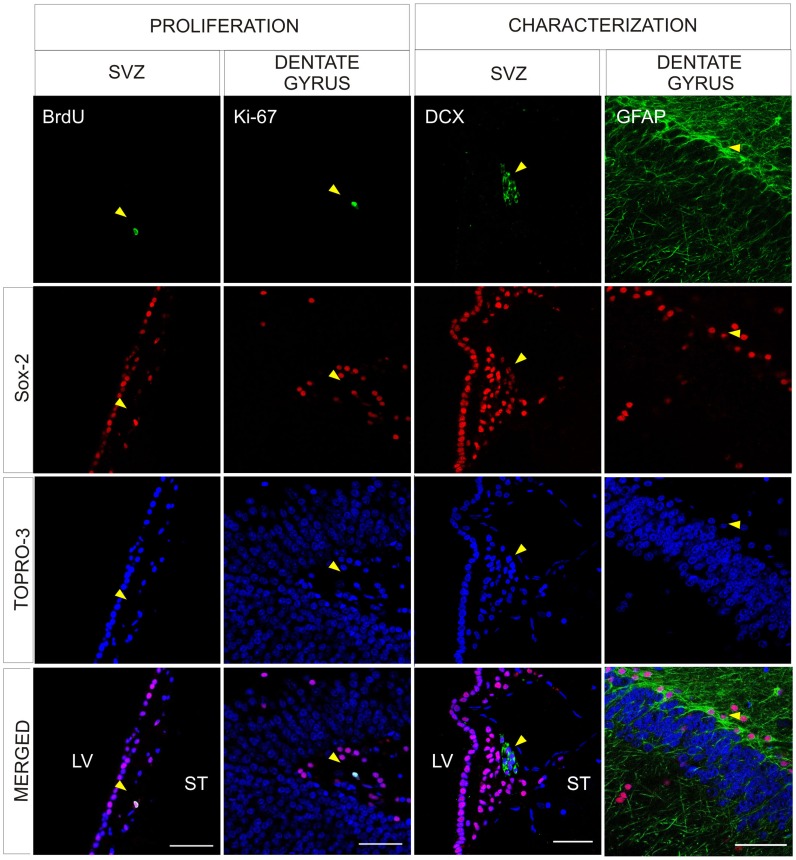
Characterization of Sox-2^+^ cells in the neurogenic niches. Confocal images showing Sox-2^+^ cells in the neurogenic niches. Representation of Sox-2^+^ cells in the SVZ that incorporate BrdU and Sox-2^+^ cells in the dentate gyrus of the hippocampus that co-express Ki67. Examples of Sox-2^+^/DCX^+^ in the SVZ and Sox-2^+^/GFAP^+^ in the SGL of the dentate gyrus. There were no differences between the control and MPTP treated animals. Scale bar = 50 µm. Abbreviations: subventricular zone: SVZ; doublecortin: DCX; subgranular layer: SGL; 1-methyl-4-phenyl-1,2,3,6 tetrahydropyridine: MPTP.

To evaluate the self-renewal capacity of Sox-2^+^ cells, we administered BrdU to control animals and sacrificed them 3 months later (see methods for details) and analyzed the co-localization of BrdU and Sox-2. We also examined the expression of Ki-67 in Sox-2^+^ cells to determine their proliferative capacity. As expected, we found double-labeled cells using both markers in the SVZ and SGZ (Fig. 2 and data not shown). Consistent with the expression of Sox-2 in stem cells and progenitors at different maturation stages, in the SVZ there were Sox-2^+^ cells that expressed DCX (Fig. 2) or GFAP (not shown). These cells were also found in the SGZ (Fig. 2 and data not shown). The intensity of Sox-2^+^ staining was weaker in the Sox-2^+^/DCX^+^ cells than in adjacent Sox-2^+^ cells (progenitor cells and transit amplifying progenitors) (Fig. S2A) [32].

**Figure 3:**
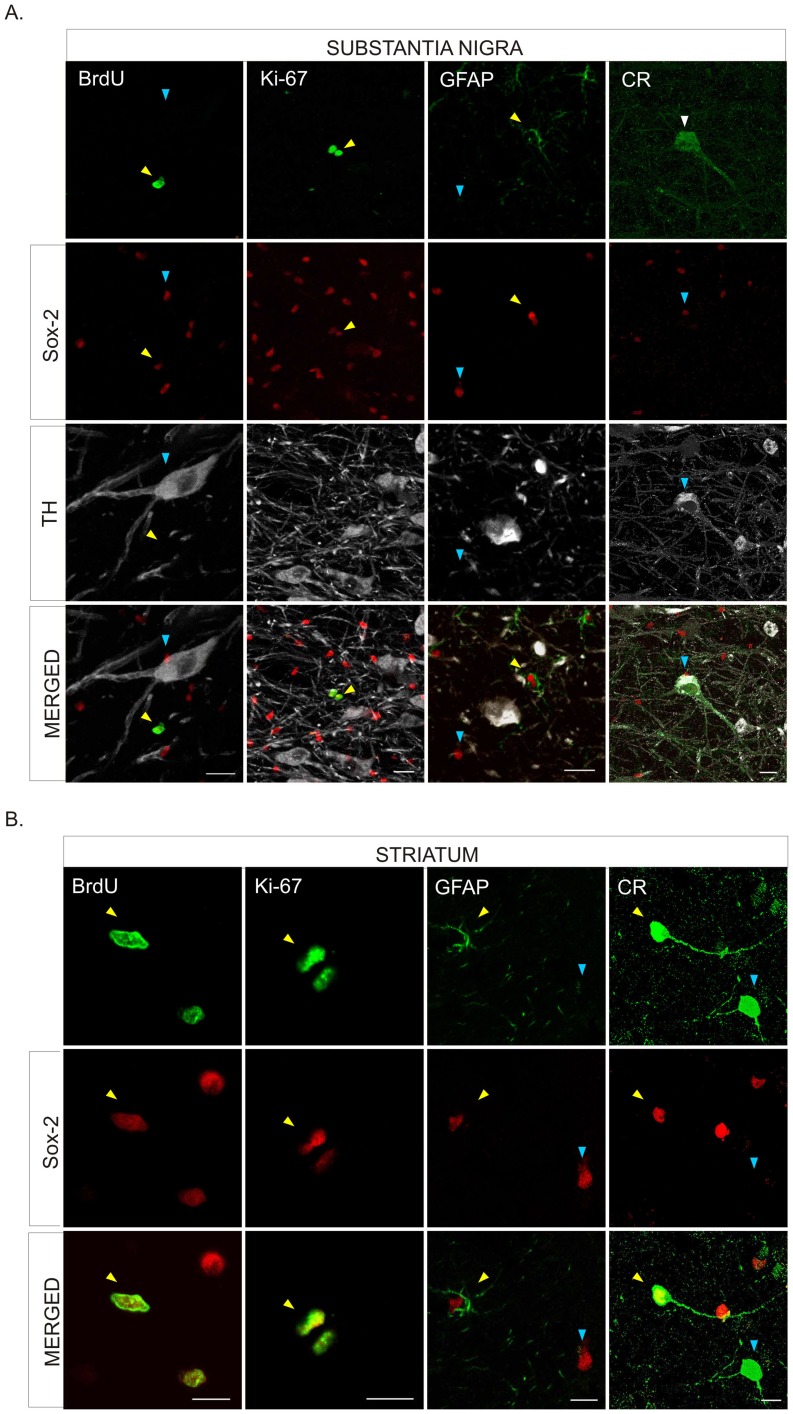
Characterization of Sox-2^+^ cells in the substantia nigra and striatum of naïve adult primates. **A.** Confocal images show BrdU incorporation (yellow arrowhead), and co-expression of Ki67 (yellow arrowhead) in Sox-2^+^ cells in the parenchyma of the SNpc. Sox-2^+^ cells were never positive for TH. Some Sox-2^+^ cells corresponded to satellite glial cells sitting on TH^+^ neurons, as shown in the merged panels (blue arrowheads). Many Sox-2^+^ cells expressed GFAP (yellow arrowhead) but there were also Sox-2^+^/GFAP^–^ (blue arrowhead) that may correspond to amplifying progenitors. There was no Sox-2^+^/CR^+^ co-expression in any cell in this region. Scale bar = 20 µm. **B.** In the striatal parenchyma some Sox2^+^ cell showed BrdU incorporation (yellow arrowhead) and co-expressed Ki67 (yellow arrowhead). Most Sox-2^+^ striatal cells expressed GFAP (yellow arrowhead) but there were also Sox-2^+^/GFAP^–^ (blue arrowhead). Some CR^+^ striatal neurons showed nuclear expression of Sox-2 (yellow arrowhead) while others did not (blue arrowhead). Scale bars = 10 µm. Abbreviations: Substantia nigra pars compacta: SNpc; tyrosine hydroxylase: TH; glial fibrillary acidic protein: GFAP; calretinin: CR.

**Figure 4:**
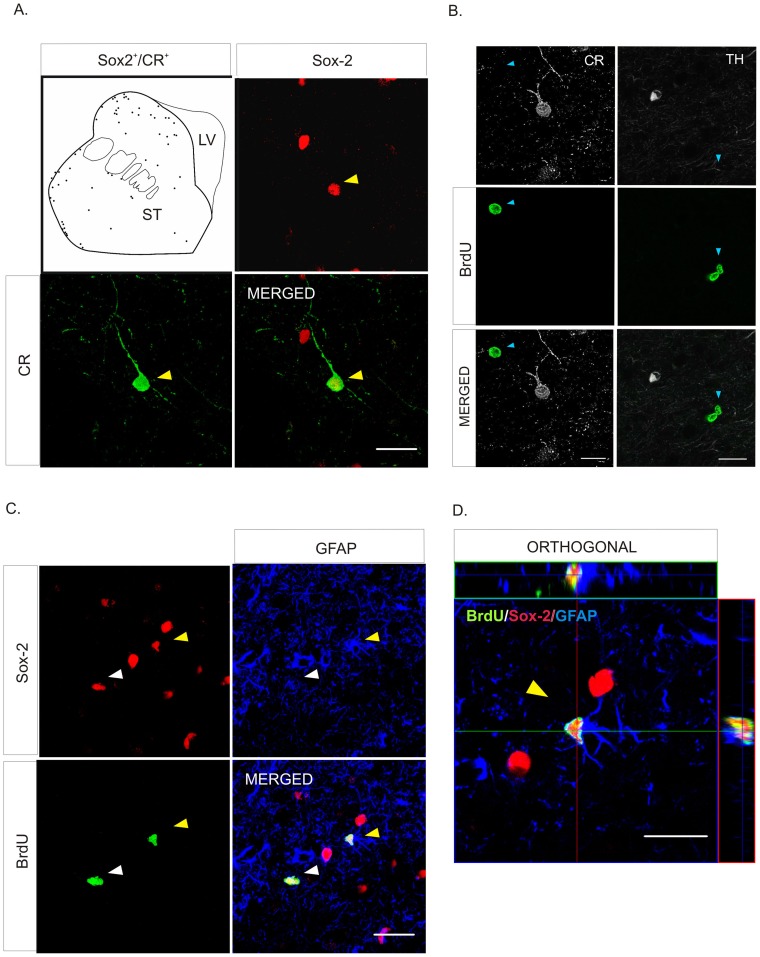
Phenotype of Sox-2 striatal cells in naïve adult primates. **A.** Schematic drawing of the distribution of Sox-2^+^/CR^+^ neurons in control animals. Note that they are located close to the dorsolateral border of the striatum; the inmunofluorescence images show the co-localization (yellow arrowhead) of Sox-2 (red) and CR (green). An orthogonal reconstruction is shown in supplemental Fig. S3A. **B.** TH^+^ striatal cells never co-localized with BrdU and the vast majority of CR^+^ striatal cells were BrdU^–^. Scale bar = 20 µm. **C.** Detailed analysis of BrdU incorporation in the striatum showed that there were some Sox-2^+^/GFAP^+^/BrdU^+^ cells (yellow arrowhead) but the majority were negative for this glial marker (white arrowhead). **D.** Orthogonal confocal reconstruction of a z-stack showing co-localization of Sox-2 (red), BrdU (green) and GFAP (blue). Scale bar = 20 µm. Abbreviations: calretinin: CR; glial fibrillary acidic protein: GFAP; tyrosine hydroxylase: TH.

Interestingly, we found Sox-2^+^ cells co-localized with BrdU and/or Ki-67 (Fig. 3A, B and Fig. S2B,C) both in the SN and the striatum of control animals, demonstrating that Sox-2^+^ cells in the nigrostriatal system of the adult primate retain proliferative capacity. Nearly all Sox-2^+^ cells were GFAP^+^ but some (less than 1%) did not express this marker (Fig. 3A, B). Indeed, we found that in the striatum some Sox-2^+^ cells expressed the neuron-specific calcium binding protein calretinin (CR) (Fig. 3B, 4A and Fig. S3A). These Sox-2^+^/CR^+^ neurons were present throughout the striatal parenchyma but were more frequently located close to the dorsolateral border of the striatum (Fig. 4A). The majority of the Sox-2^+^/CR^+^ cells had a characteristic morphology with few aspiny processes and displayed a high intensity of CR staining (Fig. 4A and Fig. S3A). Sox-2^+^/CR^+^ cells did never co-express GFAP; there were Sox-2^+^ cells that expressed neither CR nor GFAP (Fig. S3A,B). We also found that Sox-2^+^ cells did not express other markers of mature neurons such as NeuN, GAD-67 or TH (Fig. 3A and data not shown). As stated above, very few Sox-2^+^ were not GFAP^+^ and even fewer were CR^+^. However, taking into account the density of Sox-2^+^ cells and the large striatal volume, even a small percentage may have functional relevance. Interestingly, we did not find any Sox-2^+^/CR^+^ cells in the SN of control monkeys (Fig. 3A). In the striatum TH^+^ intrinsic cells were also negative for Sox-2. TH^+^ intrinsic cells were always BrdU^–^ as described [19] and CR^+^ cells were BrdU^–^ (Fig. 4B). The vast majority of BrdU^+^ -labeled striatal cells were Sox-2^+^, either BrdU^+^/Sox-2^+^/GFAP^–^ cells or BrdU^+^/Sox-2^+^/GFAP^+^ cells (Fig. 4C, D).

**Figure 5:**
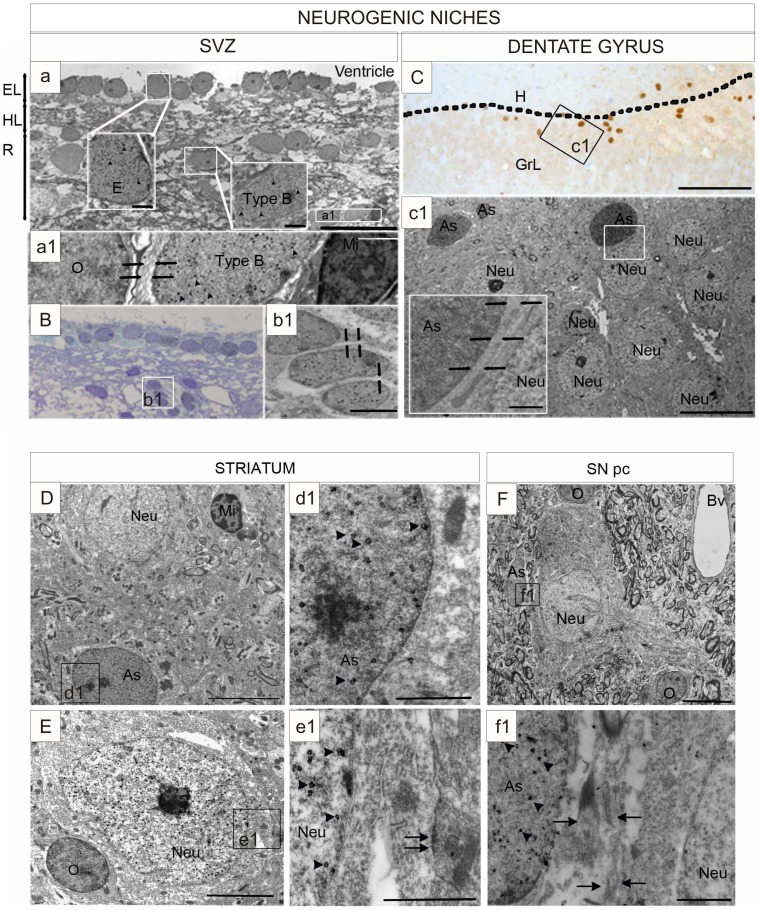
Ultra-structural analysis of Sox-2 expression. **A.** Sox-2 immuno-gold label is observed in the nucleus (arrowheads) in ependymal cells and astrocytes in ultra-thin sections of the SVZ. Boxed areas show an ependymal cell (E) and an astrocyte (As). Ependymal layer (EL), gap layer (HL), astrocyte ribbon layer (R). Scale bar = 20 um; Scale bar in boxed areas = 1 um. **a1.** Magnification shows a Sox-2^+^ astrocyte and lack of immunoreactivity in an oligodendrocyte (O) and a microglial cell (Mi). Astrocytes are recognized by the presence of dense bundles of intermediate filaments (arrows). Scale bar = 2 um. **B.** Sox-2 immuno-gold staining in a group of neuroblasts (type A cells). Scale bar = 20 um. **b1.** Magnification of three neuroblasts showing the nuclear staining. Neuroblasts form chain-like structures with characteristic intercellular spaces (arrows). Scale bar = 2 um. **C.** Light microscopy image of a semi-thin section showing the distribution and characterization of cells expressing Sox-2 in the dentate gyrus. Sox-2^+^ cells are visualized by the nuclear dark brown DAB precipitate. The section was counterstained with toluidine blue. The line marks the boundary between the granular layer (GrL) and the hilus (H) where most labeled cells were located. Scale bar = 100 um. **c1**) Neurons in the granular layer were not Sox-2^+^. Scale bar = 10 um; Insert = 1 um. **D.** Microphotograph of a striatal field of a control monkey showing a Sox-2^+^ astrocyte and unlabeled neuron (Neu) and microglial cells (Mi). Scale bar = 5 um. **d1.** Boxed area of the astrocyte (As) nucleus shows the gold particles (arrowheads). Scale bar = 1 um. **E.** A representative Sox-2^+^ neuron in the striatum of a control monkey. Scale bar = 2 um. **e1.** Boxed area shows deposition of gold particles over the nucleus (arrowheads) close to a synapse (arrow), which identifies this cell as a neuron. Scale bar = 1 um. **F.** Sox-2^+^ astrocyte in the substantia nigra pars compacta. Scale bar = 10 µm. **f1.** Boxed area shows a dense bundle of intermediate filaments (arrows) that characterizes astrocytes. Arrowheads indicate gold particles in the nucleus. Scale bar = 1 µm. Abbreviations: subventricular zone: (SVZ); 3′, 3′-diaminobenzidine: (DAB).

To verify the identity of the cells that expressed Sox-2 outside the neurogenic niches, we performed a detailed ultra-structural study using Sox-2 immuno-gold (using the neurogenic niches as reference). Sox-2 immuno-gold^+^ cells were identified by the presence of an abundant precipitate of fine gold particles distributed throughout the nuclear surface [38]. In the SVZ, both type E (ependymal cells) and type B cells (astrocyte-like) (Fig. 5A), were labeled for Sox-2. Type B cells are the progenitor cells [41]and have thick bundles of intermediate filaments in addition to glycogen granules, and dense bodies in the cytoplasm. These Sox-2^+^ progenitors were found mainly at the astrocytic ribbon layer (Fig. 5a1). Importantly, we also found migratory neuroblasts (type A cells) labeled by Sox-2^+^ (Fig. 5B). These cells were mainly located in the gap layer, had a fusiform shape and tended to form chain-like structures with characteristic intercellular spaces (Fig. 5B, 5b1). Using the same criteria in the SGZ of the dentate gyrus, we determined that Sox-2 immuno-reactive cells corresponded to the neural stem cells that share ultra-structural characteristics with astrocytes (Fig. 5C, 5c1) i.e. type B cells in the SVZ. These results corroborate that Sox-2 is expressed both by neural progenitors and neuroblasts in the neurogenic niches.

Using the same strategy, we analyzed the nigrostriatal samples. In the striatum, like in the neurogenic niches, the majority of the Sox-2 immuno-gold^+^ cells corresponded to astrocytes (Fig. 5D, 5d1). Importantly, we also observed Sox-2^+^ neurons (Fig. 5E, 5e1). These Sox-2^+^ cells were identified under the electron microscopy as likely neurons by the presence of synaptic contacts that were mostly symmetric and contained abundant pleomorphic vesicles and large mitochondria (Fig. 5e1). In the SN, all the Sox-2^+^ immuno-gold^+^ cells had morphological characteristics of astrocytes (Fig. 5F, 5f1) and we did not find any gold-labeled neurons. We never found Sox-2 nuclear immuno-reactivity in oligodendrocytes or microglial cells in any of the regions examined for this study (Fig. 5D, 5E, 5F).

### Effect of MPTP on the Sox-2^+^ Population of the Nigrostriatal System

**Figure 6:**
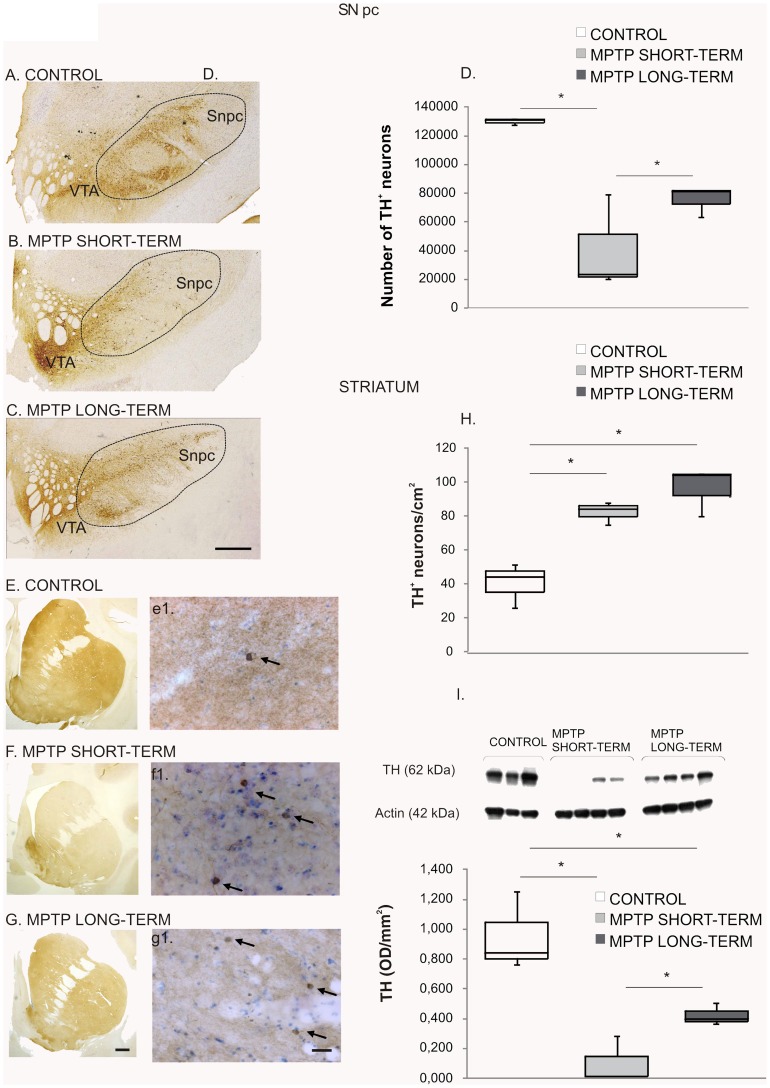
MPTP effect on the nigrostriatal system. Midbrain sections at the level of the 3rd nerve from a control (**A**), a MPTP short-term (**B**) and a MPTP long-term treated monkey (**C**) immunostained for TH. Note the severe reduction of TH immunoreactivity in the SNpc of the lesioned monkeys. Scale bar = 1 cm. **D.** Stereological quantification of TH^+^ neurons in the SNpc. Data represent median and quartiles, N = 3, *p≤0.05. Images of precommisural striatum sections from a control monkey (**E**), a MPTP short-term monkey (**F**) and a MPTP long-term monkey (**G**) immunostained for TH. Note the loss of TH^+^ fibers in the lesioned monkeys. Scale bar = 1 cm. Representative striatal sections of a control **(e’)** a MPTP short-term monkey (**f’**) and a MPTP long-term monkey (**g’**), at higher magnification showing the intrinsic TH^+^ neurons (arrows). Scale bar = 300 µm. **H.** Quantification of striatal TH^+^ neurons. There was a significant increase in the density of TH^+^ neurons after MPTP administration in both groups. Data represent median and quartiles, N = 3, *p≤0.05. **I.** Western blot to assess striatal TH levels showing a reduction in the MPTP-short term group and a partial recovery in the MPTP-long term group with respect to controls. Data represent median and quartiles, N = 3, *p≤0.05. Abbreviations: 1-methyl-4-phenyl-1,2,3,6 tetrahydropyridine: MPTP; tyrosine hydroxylase: TH; substantia nigra pars compacta: SNpc.

To investigate the effects of dopamine denervation on the Sox-2^+^ cell population, we used MPTP administration and examined the brains 3 months (short-term) and 18 months (long-term) after the lesion. Quantification of TH^+^ neurons was performed on the right SN and revealed a significant loss of dopamine neurons in all MPTP-treated monkeys (Fig. 6A, B, C, D); control group: Q1∶129246; median: 130602; Q3∶131175 TH^+^ neurons, *vs.* MPTP short-term group: Q1∶21913; median: 23367; Q3∶51191 TH^+^ neurons, p = 0.034 and *vs.* MPTP long-term group: Q1∶72495; median: 81373; Q3∶81395 TH^+^ neurons, p = 0.034. The loss of nigral dopamine neurons was higher in the MPTP short-term group (67%) than in the MPTP long-term group (40%), p = 0.043 (Fig. 6D). There was a relative preservation of the neuronal TH^+^ population of the ventral tegmental area (VTA) in both groups (Fig. 6A, B, C). Since the loss of TH neurons was less severe in the MPTP long-term animals than in the MPTP short-term animals we evaluated the striatal levels of TH by western blot in all groups (control group: Q1∶0,801; median: 0,840; Q3∶1,047 OD/mm^2^, *vs.* MPTP short-term group: Q1∶0,010; median: 0,012; Q3∶0,148 OD/mm^2^, p = 0.034 and *vs.* MPTP long-term group: Q1∶0,382; median: 0,397; Q3∶0,451 OD/mm^2^, p = 0.077. MPTP short-term group *vs.* MPTP long-term group, p = 0.021) (Fig. 6I). These data showed that the levels of TH were significantly lower in the short-term animals suggesting that there was a partial recovery in the long-term group. This is also consistent with the stereological counts in the SN.

We found, as previously described [19], [42]that MPTP-lesioned animals showed a significant increase in the number of intrinsic striatal TH^+^ neurons; control group: Q1∶35; median: 44; Q3∶48 (neurons/cm^2^) *vs.* MPTP short-term group: Q1∶80; median: 84; Q3∶86 (neurons/cm^2^), p = 0.034 and *vs.* MPTP long-term group: Q1∶92; median: 104; Q3∶105 (neurons/cm^2^), p = 0.034 (Fig. 6H). However, the number of striatal TH^+^ neurons was not different between the two groups of MPTP-monkeys (p = 0.083) (Fig. 6E, e1, F, f1, G, g1, H). These data show that, in spite of the partial compensation observed in TH levels, the increase in TH^+^ striatal cells remained stable for the duration of the study (18 months). The TH^+^ striatal neurons were distributed in a typical pattern [43] being especially abundant in the rostro-dorsal region of the caudate nucleus and putamen (Fig. S4A). The majority of TH^+^ cells were bipolar neurons with a round or oval perikaryon [43], [44].The distribution and the morphology were similar in control and MPTP-lesioned animals (Fig. S4A).

**Figure 7:**
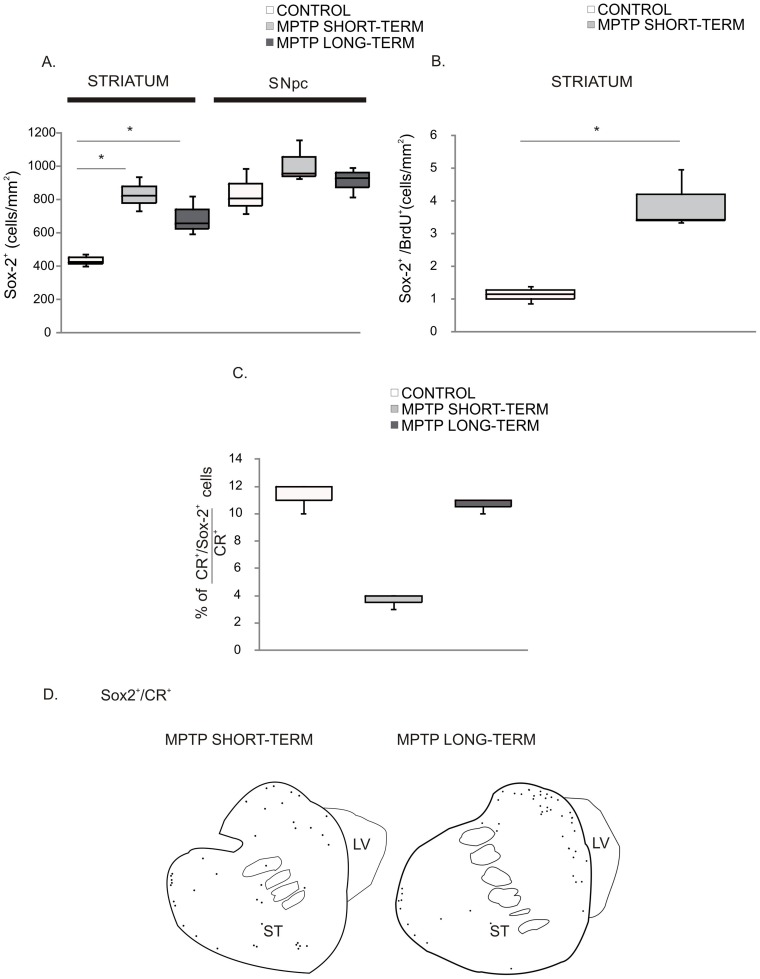
MPTP effect on Sox-2^+^ cells of the nigrostriatal system. **A.** Stereological estimation of Sox-2^+^ cells in the SNpc and the striatum of the different groups of animals showed the significant increase of Sox-2^+^ cells in the striatum of MPTP-treated monkeys in both groups. Data represent median and quartiles, N = 3, *p≤0.05. **B.** There was an increase in the density of BrdU^+^/Sox-2^+^ cells in the MPTP-short-term group respect to controls. Data represent median and quartiles, N = 3, *p≤0.05. **C.** There were Sox-2^+^/CR^+^ cells both in control and in MPTP treated animals but quantification showed a significant reduction of this population in the MPTP short-term group that was no longer present in the MPTP long-term group Data represent median and quartiles, N = 3, *p≤0.05. **D.** Schematic drawing of the distribution of CR^+^ cells in the MPTP treated animals. Note that they show the same distribution as controls (see Fig. 4A) but the density appears lower in the short-term group. Abbreviations: substantia nigra pars compacta: SNpc; 1-methyl-4-phenyl-1,2,3,6 tetrahydropyridine: MPTP; calretinin: CR.

The regional distribution of Sox-2^+^ cells was not different between control and MPTP-treated animals, but the density of Sox-2^+^ cells appeared to be higher in MPTP-treated animals as compared with controls. Indeed, quantification of Sox-2^+^ cells (Fig. 7) confirmed that, in the striatum, the density was higher in the MPTP-treated groups than in controls (control group: Q1∶424; median: 436; Q3∶458 cells/mm^2^, *vs*. MPTP short-term group: Q1∶756; median: 798; Q3∶849 cells/mm^2^, p = 0.05; MPTP long-term group: Q1∶617; median: 645; Q3∶720, p = 0.05). Both groups of MPTP-monkeys exhibited similar density of striatal Sox-2^+^ cells (Fig. 7A). In contrast, in the SN the density of Sox-2^+^ cells was similar in the 3 groups (control group: Q1∶740; median: 783; Q3∶862 cells/mm^2^, *vs.* MPTP short-term group: Q1∶903; median: 915; Q3∶1007 cells/mm^2^, p = 0.275 and *vs.* MPTP-long-term group: Q1∶840; median: 890; Q3∶920 cells/mm^2^, p = 0.275; MPTP short-term group *vs.* MPTP-long-term group, p = 1) (Fig. 7A). We did not observe any differences between the controls and the MPTP-treated animals regarding ultra-structural features or phenotype of Sox-2^+^ cells. However, we found a significant increase in the density of BrdU^+^/Sox-2^+^ cells in the MPTP-lesioned animals (control group: Q1∶1.01; median: 1.16; Q3∶1.27 cells/mm^2^, *vs.* MPTP-short-term group Q1∶3.40; median: 3.44; Q3∶4.20 cells/mm^2^, p = 0.034) (Fig. 7B) in the striatum.

As described above for control animals, in addition to astrocytes there were Sox-2^+^/CR^+^ neurons in the striatum of the MPTP-treated animals. Thus, to evaluate whether the neurogenic potential of striatal Sox-2^+^ cells was changed by MPTP, we quantified the percentage of striatal CR^+^ cells that co-expressed Sox-2. We found that in the control group 11% of CR^+^ cells were Sox-2^+^. Remarkably, the number of CR^+^/Sox-2^+^ cells was significantly reduced in the MPTP short-term group (control group: Q1∶11; median: 12; Q3∶12% *vs.* MPTP short-term group: Q1∶3.5; median: 4; Q3∶4%, p = 0.05). In contrast, in the MPTP long-term animals (examined 18 months after MPTP) the percentage of CR^+^ that co-expressed Sox-2 was the same as in control animals (MPTP long-term group: Q1∶10.5; median: 11; Q3∶11%) (Fig. 7C). These results suggest that MPTP caused a temporary disruption in the neurogenic capacity of Sox-2^+^ striatal cells. As shown in Fig. 7D, the distribution of Sox-2^+^/CR^+^ neurons was not changed (compare with Fig. 4A) in spite of the severe reduction of this population in the short-term group. Note that the distribution of Sox-2^+^/CR^+^ cells was remarkably similar to that found for the TH^+^ cells that increase in the striatum after MPTP administration (Fig. S4A).

## Discussion

In this work we demonstrate that Sox-2^+^ cells with self-renewal capacity are present in the striatum and the SN of the adult primate brain. Importantly, although the majority of Sox-2^+^ cells in the nigrostriatal system were GFAP^+^, we found that some Sox-2^+^ cells were GFAP^–^ (and may thus correspond to neural amplifying progenitors, like in the SGZ). Furthermore, in the striatum but not in the SN, we found that some CR^+^ neurons expressed Sox-2, suggesting that there is a population of Sox-2^+^ striatal cells capable to differentiate into neurons *in vivo* in the adult primate brain.

**Figure 8:**
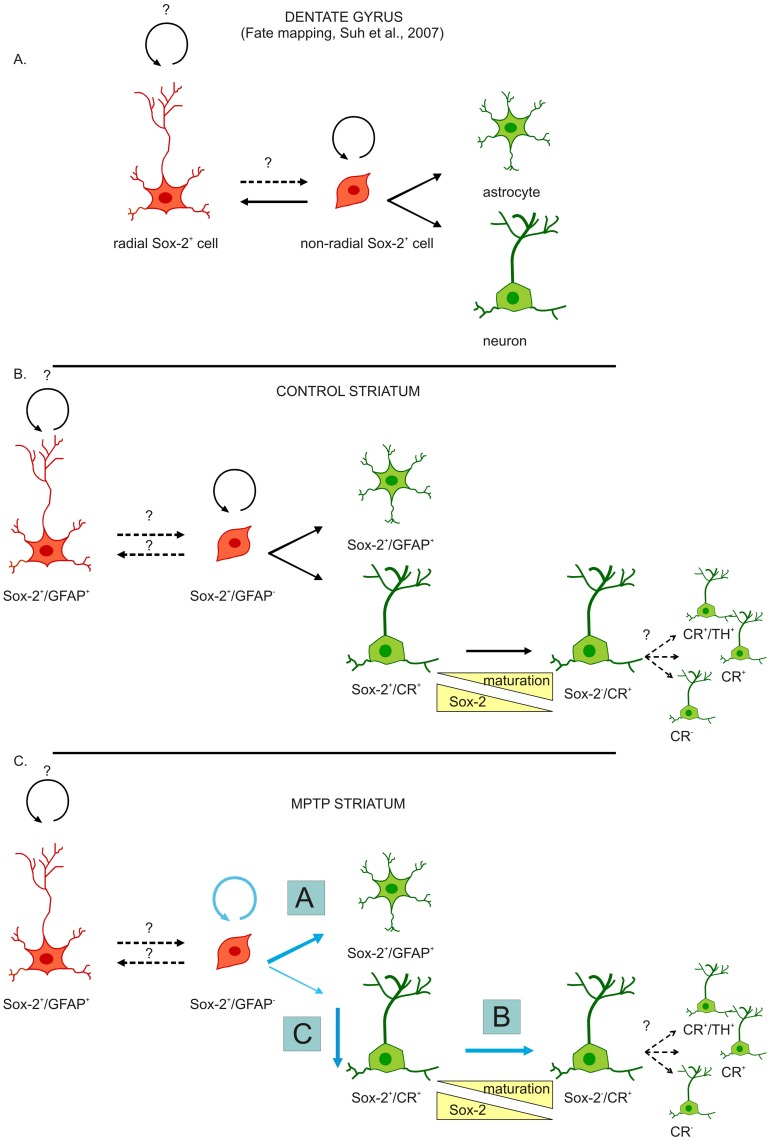
Cell renewal and differentiation dynamics of progenitors in the striatum. Schematic representation of hypothetic dynamics of endogenous striatal progenitors and the effects of MPTP. **A.** For reference we represented on top, the results of fate mapping of Sox-2^+^ cells in the murine dentate gyrus showing that non-radial Sox-2^+^ progenitors have self-renew potential and give rise to both neurons and astrocytes [26]. **B, C.** Similarly, we propose than in the normal situation, Sox-2^+^ striatal progenitors (Sox-2^+^/GFAP^−/^CR^–^) divide to generate new Sox-2^+^ progenitors. Moreover, some Sox-2^+^ progenitors give rise to astrocytes and (few) Sox-2^+^/CR^+^ neurons that after several maturation steps could switch off Sox-2 expression and give rise to different mature phenotypes (including TH^+^). Following MPTP administration the number of Sox-2^+^ progenitors increases while the number of CR^+^ cells that express Sox-2 is decreased. Possible mechanisms are A) MPTP favours gliogenesis from Sox2 progenitors at the expense of neurogenesis. B) MPTP (or lack of dopamine) accelerates maturation of Sox-2^+^/CR^+^ cells. C) Sox-2^+^/CR^+^ cells are susceptible to MPTP toxicity. Abbreviations: 1-methyl-4-phenyl-1,2,3,6 tetrahydropyridine: MPTP; calretinin: CR; tyrosine hydroxylase: TH.

Sox-2 is essentially absent in mature neurons in the adult brain [31], [32]. Most Sox-2^+^ cells in the brain are astrocytes, which may be similar to neural stem cells in terms of potentiality but quiescent, and susceptible to be activated by injury or other stimuli [45]. In adult tissues, stem cells are in different states of dormancy and may have different roles in tissue homeostasis and regeneration. Sox-2 is present in stem cells and neural progenitors and is important to inhibit cell cycle exit and neuronal maturation. Nevertheless, it has been described [32] that in cells in the germinal niches weak Sox-2 co-localizes with early neuronal markers, indicating a concomitant appearance of neuronal phenotypic markers, i.e. CR, with decreasing Sox-2 levels [46]. In contrast with a previous study in rodents [31], concluding that Sox-2^+^ cells lack proliferative capacity outside the adult neurogenic niches, we found that Sox-2^+^ cells in the adult primate SN and striatum retained mitotic capacity under baseline conditions. This discrepancy is likely due to methodological differences in BrdU administration and time of analyses. The analyses of BrdU incorporation showed that Sox-2^+^ cells can self-renew and give rise mainly to GFAP^+^ astrocytes. Sox-2^+^ cells in the nigrostriatal regions were similar to those in the neurogenic niches of the SVZ and SGZ, if considerably less abundant. This is consistent with experiments performed in rodents, in which in cultures of an equal mass of neurogenic and non-neurogenic areas of the adult brain, neurogenic zones yield a higher proportion of progenitor-like colonies [10]. Fate map analysis of Sox-2^+^ cells in the dentate gyrus has revealed that non-radial Sox-2^+^ cells, are neural progenitors with self- renewal potential that give rise to both neurons and astrocytes [28](Fig. 8).

CR is a neuron-specific calcium binding protein that is expressed in a subset of mature striatal interneurons [46]. However, it is worth noting that CR is also expressed in neuroblasts and young neurons during embryonic development [47] and in the adult neurogenic areas in hippocampus [48], [49] and rostral migratory stream [50]. In the DG, CR is regarded as a transitional maturation marker for new granule cells (expressed before they become calbindin^+^ neurons) [48], [51], [52]. We propose here that striatal Sox-2^+^/CR^+^ cells are immature because Sox-2 is still detectable. In the hippocampus and the SVZ weak immuno-reactivity for Sox-2 coincides with expression of DCX and other neuronal markers, strongly suggesting that there is an intermediate stage in which young neurons co-express Sox-2 (going down) and a neuronal marker (going up). In spite of several reports describing newborn neurons in the striatum as CR^+^ interneurons [14], [53], we think that, given the transient character of CR expression during various developmental and maturation stages, it is not possible to establish whether the expression of CR in these neurons is permanent or transient. Because complete maturation of neurons requires down-regulation of Sox-2 [24], [25] we can only speculate about the final fate of these striatal newborn neurons. Therefore, in our study we cannot determine whether CR indicates the final phenotype of new striatal neurons, or a transient stage in maturation (Figure 8B, C).

Interestingly, MPTP caused a marked increase in the number of Sox-2^+^ cells in the striatum, but not in the SN, and an increase in the amount of newly generated Sox-2^+^/BrdU^+^ striatal cells. Previous studies in rodents have shown increased numbers of neural progenitors in the striatum and midbrain after MPTP administration [54], [55]. However, while in the striatum the newly generated cells persisted for a long period of time (as astrocytes), in the SN the newborn cells maintained an undifferentiated phenotype and showed short survival times [54], [55]. We failed to detect any increase in Sox-2^+^ cells in the SN, but cannot completely rule out a transient increase of short-lived Sox-2^+^ cells in the region– that were no longer present at the time of analysis (3 months after the MPTP administration). Taken together, our results point out that MPTP, either directly or indirectly, stimulates the renewal of Sox-2^+^ cells in the striatum and suggest a different potential and/or reactivity of Sox-2^+^ cells in the two regions. However, this can also be due to differences in the microenvironment as both the direct consequences of the MPTP lesion (most conspicuously the abrupt fall of dopamine levels in the striatum) and perhaps some compensatory mechanisms, differ between the two regions [54]. Indeed, dopamine has long been implicated in the control of adult neurogenesis in the SVZ [56], [57], [58].

In contrast with the reactive neurogenesis that has been shown to occur in the primate sensorimotor system after injury [20] and in the caudate nucleus and cortex after stroke [21], here we found that MPTP markedly decreased the percentage of Sox-2^+^/CR^+^ cells in the striatum. Intriguingly, this effect was reversible and by 18 months there was a complete normalization of Sox-2^+^/CR^+^ numbers. It has been previously reported that aged monkeys treated with MPTP showed, five weeks after the last injection, a reduced formation of neuroblasts in the SVZ [57]. Therefore, it is possible that the acute reduction in Sox-2^+^/CR^+^ striatal neurons that we observed is a reflection of a more general effect on neurogenesis by MPTP (or by the fall in dopamine levels).

Alternatively, we have to consider the possibility that Sox-2^+^/CR^+^ cells are susceptible to the neurotoxic effects of MPTP. Although a direct effect is unlikely, a decrease in growth factors or morphogens locally released by dopamine axons could be involved in this effect. Such an effect could explain the temporary reduction in the percentage of Sox-2^+^/CR^+^3 months after MPTP.

Another tantalizing possibility (if speculative, at this time) is that there is a link between the robust increase in TH^+^ intrinsic striatal neurons and the decrease in Sox-2^+^/CR^+^cells. We have previously reported that a proportion of striatal intrinsic TH^+^ neurons co-express CR in the primate [44]. Sox-2^+^/CR^+^ cells could represent an immature stage of this reactive population that appears rapidly following dopamine lesions. Upon down-regulation of Sox-2, these neurons would up-regulate TH (becoming CR^+^/TH^+^), and this transition could be accelerated by the reduction of dopamine levels caused by MPTP. This could account for both the increase in TH^+^ cells and the reduction in the percentage of Sox-2^+^/CR^+^ cells shortly after MPTP administration. Once the dopamine levels are partially restored and the insult is over, new Sox-2^+^/CR^+^ cells would be generated from local Sox-2^+^ progenitors to restore the pool of Sox-2^+^/CR^+^ cells that exists in physiological conditions (Fig. 8). In the future, lineage-tracing experiments in the mouse could help determine the relationship between both populations. In any event, given their neurogenic capacity, reactivity and plasticity after a neurotoxic insult in the primate, the striatal Sox-2^+^ cells represent an attractive target for endogenous repair strategies.

Notwithstanding, we have to point out several potential caveats in our study. First, although BrdU is considered a reliable method to identify newborn cells in the brain, there are few studies in primates. We have chosen this regime of administration of BrdU based on previous studies in the primate brain [6], [36], [37], [59] suggesting that the dose and time are sufficient to detect newborn mature neurons in the dentate gyrus and neocortex. However, we cannot rule out the possibility that more time is necessary to detect newborn mature neurons originated in the striatum or migrating from the SVZ. Moreover, Cameron and McKay (2001) [55], systematically studied the effect of higher doses of BrdU in adult rats. They found that doses up to 600 mg/kg did not have adverse effects on either the animals or the labeled cells. Furthermore, the number of BrdU-labeled cells in the dentate gyrus markedly increased between doses of 50 mg/kg and 300 mg/kg. At the higher dose they estimated that 9,000 new cells were added to the dentate gyrus every day, most of which became neurons. Although little is known about the similarity of rats and macaques regarding the clearance of BrdU and the ability of BrdU to cross the blood-brain barrier, these results raise the possibility that in our study we underestimated the numbers of new adult-generated cells in the macaque striatum.

Regarding the MPTP primate model, major limitations are the toxic nature of the lesion, the lack of progression and the absence of alpha-synuclein cytoplasmic inclusions [60].Therefore, the compensatory or regenerative mechanisms induced by MPTP can be radically different from those occurring in clinical Parkinson’s disease. Nevertheless, in our paradigm (low-dose repeated systemic administration of the toxin) both the loss of dopamine neurons in the SN and the decrease in striatal dopamine content are gradual, providing a closer approximation to Parkinson's disease progressive features [61]. Our data cannot indicate whether neurogenesis takes place in the human striatum but our findings showing a unique temporal response of the striatal Sox-2^+^/CR^+^ population in MPTP-treated monkeys merit further investigation.

Finally, both Sox-2 and CR expression are dynamically regulated during developmental and adult neurogenesis [24], [25], [48], [49], [50], [52]. This further increases the complexity of the analyses of their respective progenies. For this reason, we have proposed alternative models that fit our results, understanding that our data alone cannot determine the final destiny of these populations (that will require fate-mapping experiments).

In conclusion, we demonstrate here that progenitor cells with neurogenic and proliferative potential persist in the normal adult primate striatum. The response of this population to an injury suggests that they are amenable to extrinsic manipulation and therefore good candidates for regenerative and repair therapies. Elucidating the signals and factors that regulate fate decisions in Sox-2^+^ striatal progenitor cells can pave the way for endogenous restoration of dopamine transmission in Parkinson's disease.

## Supporting Information

Figure S1 **A.** Confocal images showing no staining for Sox-2 in absence of the primary antibody against Sox-2 (negative control of Sox-2 staining). Scale bar = 20 µm. **B.** Confocal images and an orthogonal confocal reconstruction of a z-stack showing nuclear localization of Sox-2 in the SN (the same nuclear staining was found in the striatum and in neurogenic niches). Scale bar = 100 µm. **C.** To corroborate the density of Sox-2+ cells obtained by stereology in the SN, we counted the number of Sox-2+ cells in LSM frames (225 µm×225 µm) using ImageJ software. A representative image is shown with 39 markers in 0.050625 mm^2^ which is roughly equivalent to our results (770/mm^2^) using more precise stereological methods. Scale bar = 20 µm.
(TIF)

Figure S2 **A.** Double immunofluorescence images showing some Sox-2^+^/DCX^+^ cells in the SVZ. Note the weaker staining of Sox-2^+^/DCX^+^ cells comparing with other Sox-2^+^ cells of the SVZ (stem cells and transit amplifying progenitors). Scale bar = 20 µm. **B, C** Double immunofluorescence images and orthogonal confocal reconstructions of z-stacks showing Sox-2^+^ cells positive for BrdU and Ki-67. The images shown were taken from the SN. Equal results were obtained from double-labeled cells in the striatum (not shown). Scale bar = 10 µm. Abbreviations: doublecortin: DCX.
(TIF)

Figure S3 **A.** Orthogonal confocal reconstruction of a z-stack showing a CR^+^ striatal cell (green) co-localized with Sox-2 (red). Separate channels are shown in Fig. 4A. Scale bar = 20 µm. **B.** Triple immunofluorescence images showing that some Sox-2^+^ cells were negative for GFAP and CR (blue arrowhead). Scale bar = 20 µm. Abbreviations: calretinin: CR; glial fibrillary acidic protein: GFAP.
(TIF)

Figure S4 **A.** Schematic drawing of the distribution of TH^+^ neurons in control animals. Note that they are located close to the dorsolateral border of the striatum and the distribution is similar to that of Sox-2^+^/CR^+^ cells. **B.** Orthogonal confocal reconstruction of a z-stack showing a TH^+^ striatal cell (green) co-localized with calretinin (CR) (red). Scale bar = 20 µm. Abbreviations: tyrosine hydroxylase: TH; calretinin: CR.
(TIF)
