# Correction: Sox-2 Positive Neural Progenitors in the Primate Striatum Undergo Dynamic Changes after Dopamine Denervation

**DOI:** 10.1371/annotation/e844adbf-7acf-4b7a-8eaa-46e356c5d9f5

**Published:** 2013-09-20

**Authors:** Cristina Ordoñez, Paz Moreno-Murciano, Maria Hernandez, Carla Di Caudo, Iñaki-Carril Mundiñano, Nerea Vazquez, Jose Manuel Garcia-Verdugo, Rosario Sanchez-Pernaute, Maria-Rosario Luquin

The name of the fifth author was given incorrectly. The correct name is: Iñaki-Carril Mundiñano. 

The correct citation is: Ordoñez C, Moreno-Murciano P, Hernandez M, Di Caudo C, Mundiñano I-C, et al. (2013) Sox-2 Positive Neural Progenitors in the Primate Striatum Undergo Dynamic Changes after Dopamine Denervation. PLoS ONE 8(6): e66377. doi:10.1371/journal.pone.0066377. 

The correct abbreviation in the Author Contributions statement is: I-CM. 

