# Supplementary figures and images for: Sox-2 Positive Neural Progenitors in the Primate Striatum Undergo Dynamic Changes after Dopamine Denervation
Source: PLoS One. 2013 Jun 18;8(6):e66377. doi: 10.1371/journal.pone.0066377 (PMC3688912; doi:10.1371/journal.pone.0066377)

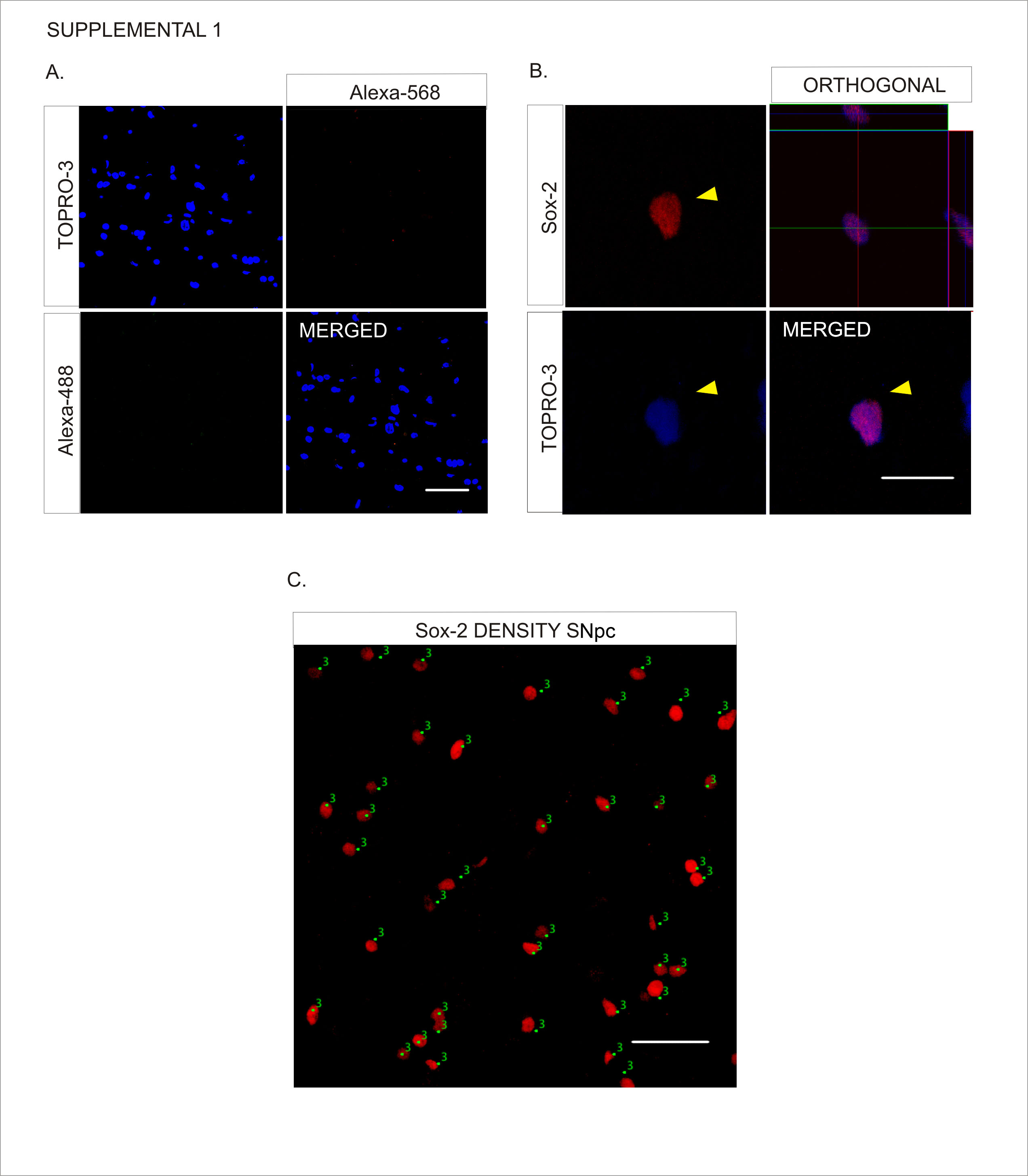

Supplement: Figure S1 — A. Confocal images showing no staining for Sox-2 in absence of the primary antibody against Sox-2 (negative control of Sox-2 staining). Scale bar = 20 µm. B. Confocal images and an orthogonal confocal reconstruction of a z-stack showing nuclear localization of Sox-2 in the SN (the same nuclear staining was found in the striatum and in neurogenic niches). Scale bar = 100 µm. C. To corroborate the density of Sox-2+ cells obtained by stereology in the SN, we counted the number of Sox-2+ cells in LSM frames (225 µm×225 µm) using ImageJ software. A representative image is shown with 39 markers in 0.050625 mm2 which is roughly equivalent to our results (770/mm2) using more precise stereological methods. Scale bar = 20 µm. (TIF) [file pone.0066377.s001.tif]

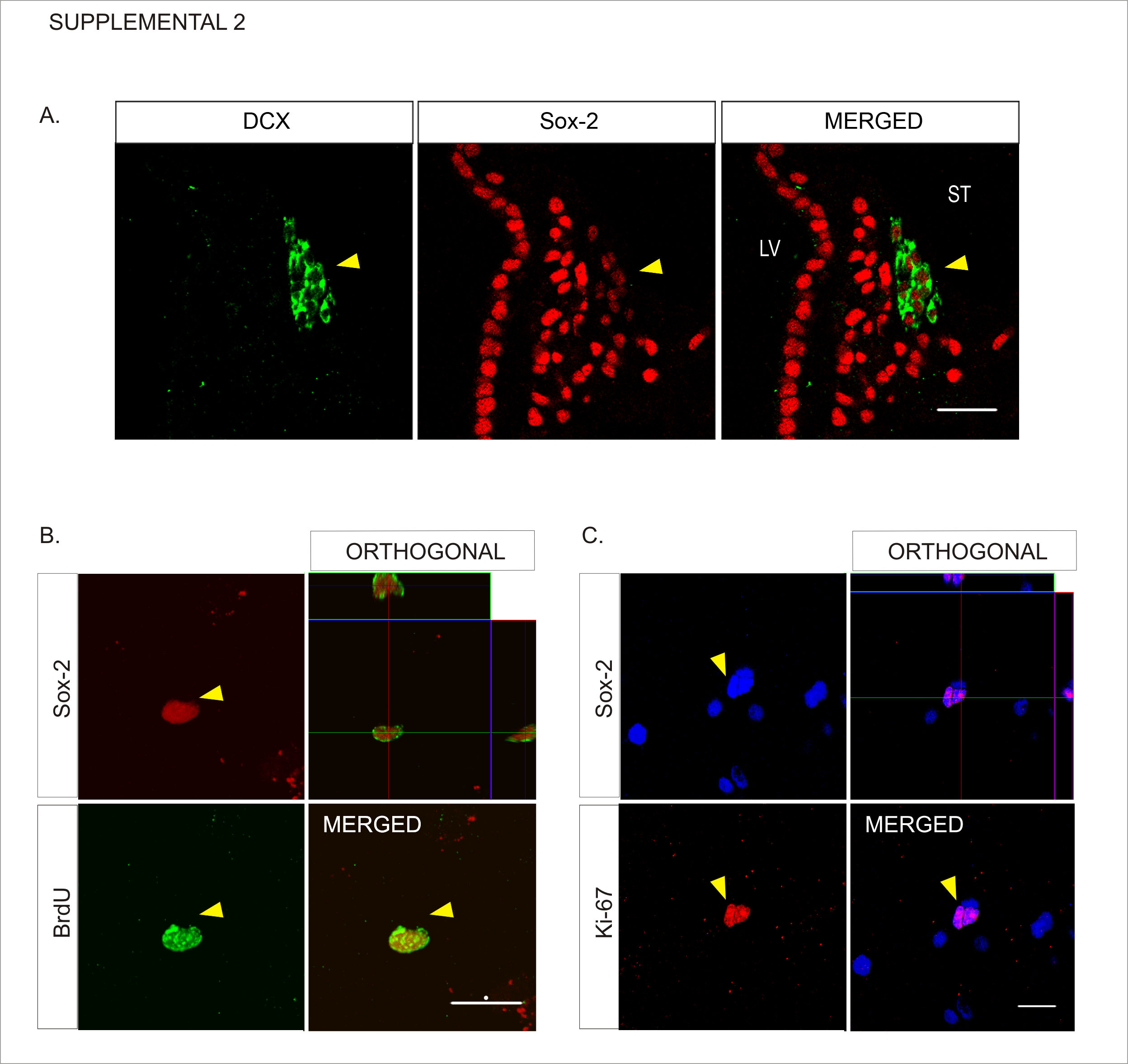

Supplement: Figure S2 — A. Double immunofluorescence images showing some Sox-2+/DCX+ cells in the SVZ. Note the weaker staining of Sox-2+/DCX+ cells comparing with other Sox-2+ cells of the SVZ (stem cells and transit amplifying progenitors). Scale bar = 20 µm. B, C Double immunofluorescence images and orthogonal confocal reconstructions of z-stacks showing Sox-2+ cells positive for BrdU and Ki-67. The images shown were taken from the SN. Equal results were obtained from double-labeled cells in the striatum (not shown). Scale bar = 10 µm. Abbreviations: doublecortin: DCX. (TIF) [file pone.0066377.s002.tif]

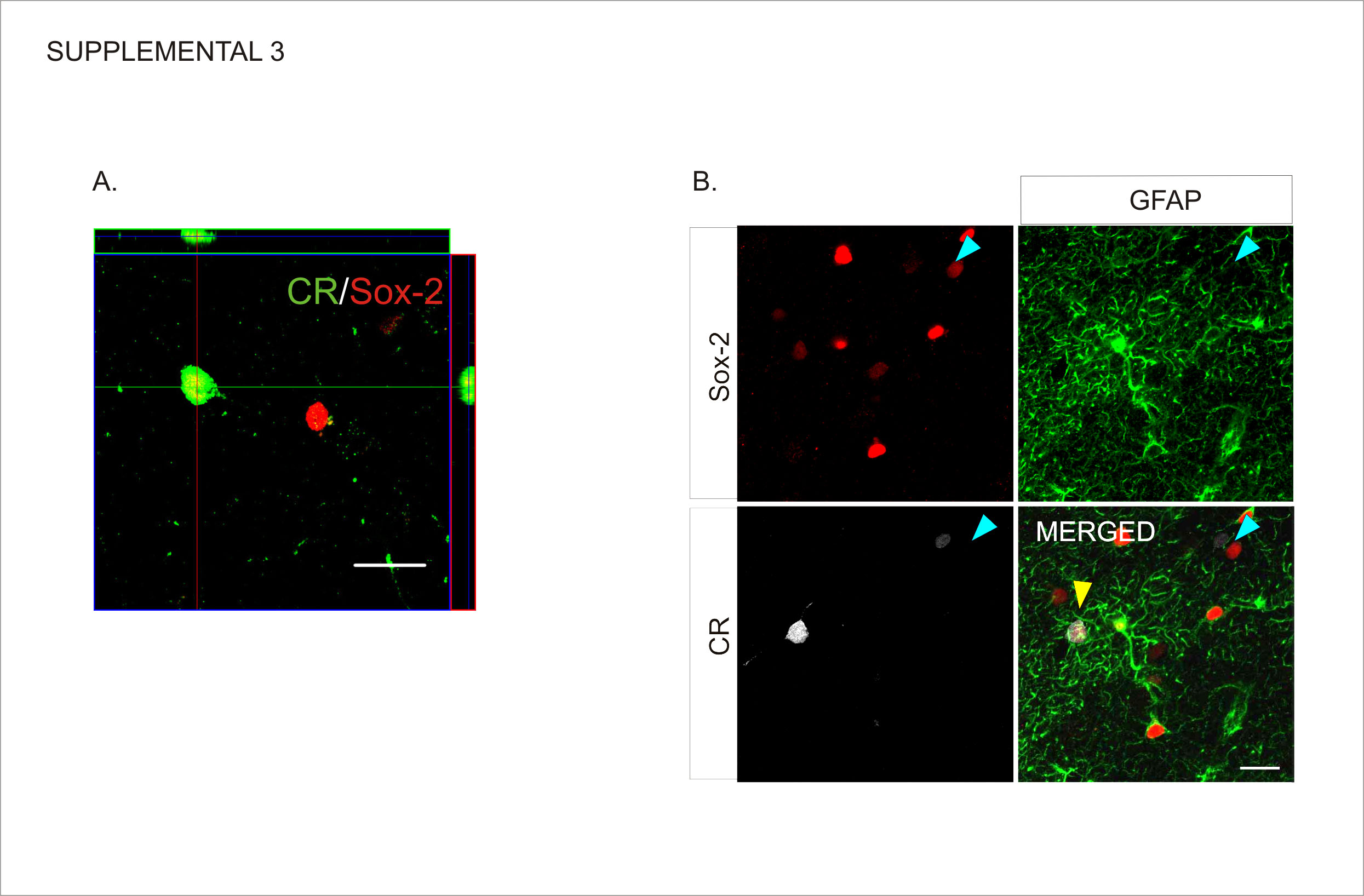

Supplement: Figure S3 — A. Orthogonal confocal reconstruction of a z-stack showing a CR+ striatal cell (green) co-localized with Sox-2 (red). Separate channels are shown in Fig. 4A. Scale bar = 20 µm. B. Triple immunofluorescence images showing that some Sox-2+ cells were negative for GFAP and CR (blue arrowhead). Scale bar = 20 µm. Abbreviations: calretinin: CR; glial fibrillary acidic protein: GFAP. (TIF) [file pone.0066377.s003.tif]

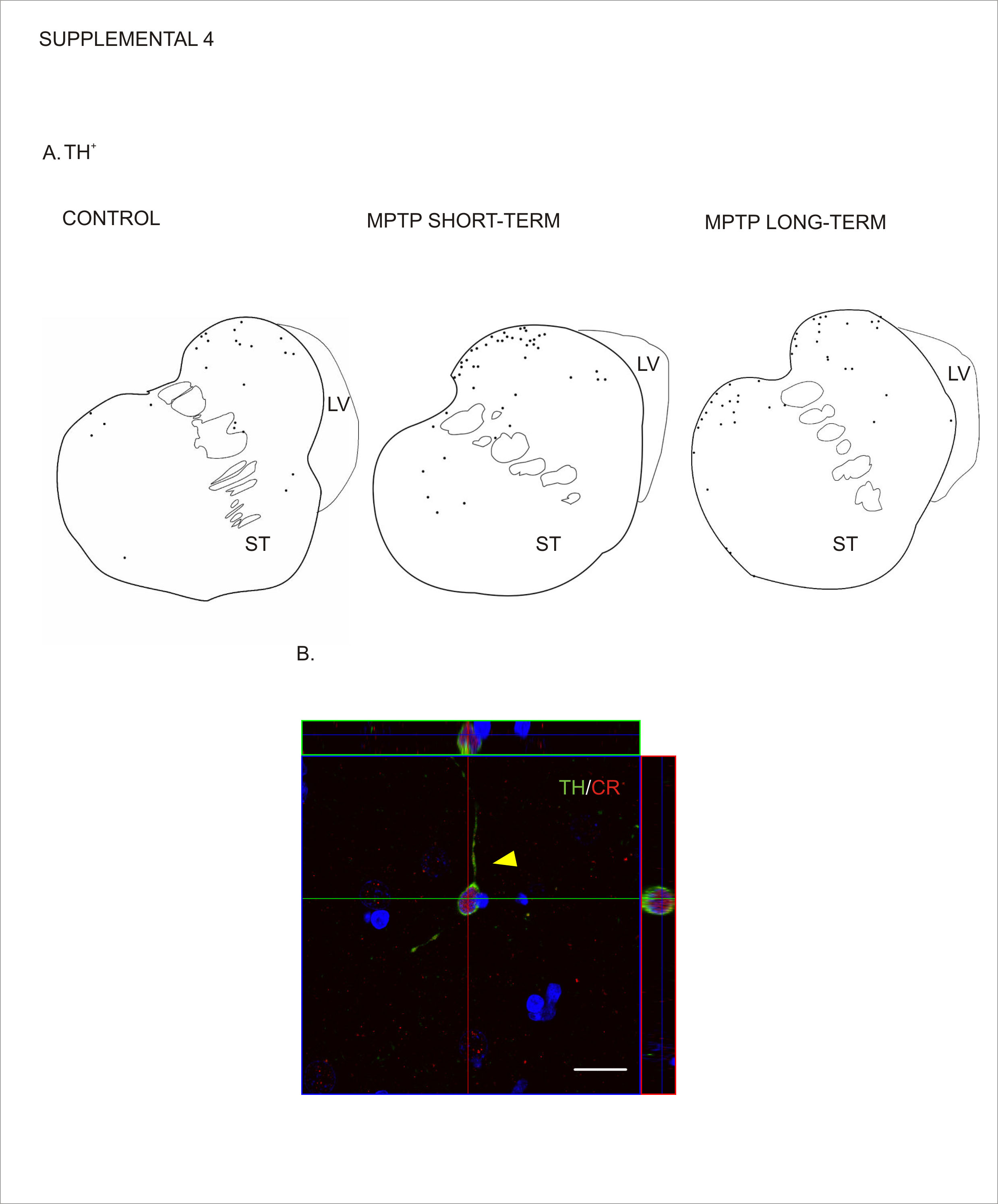

Supplement: Figure S4 — A. Schematic drawing of the distribution of TH+ neurons in control animals. Note that they are located close to the dorsolateral border of the striatum and the distribution is similar to that of Sox-2+/CR+ cells. B. Orthogonal confocal reconstruction of a z-stack showing a TH+ striatal cell (green) co-localized with calretinin (CR) (red). Scale bar = 20 µm. Abbreviations: tyrosine hydroxylase: TH; calretinin: CR. (TIF) [file pone.0066377.s004.tif]
